# Drosophila Interspecific Hybridization Causes a Deregulation of the piRNA Pathway Genes

**DOI:** 10.3390/genes11020215

**Published:** 2020-02-19

**Authors:** Víctor Gámez-Visairas, Valèria Romero-Soriano, Joan Martí-Carreras, Eila Segarra-Carrillo, Maria Pilar García Guerreiro

**Affiliations:** 1 Grup de Genòmica, Bioinformàtica i Biologia Evolutiva, Departament de Genètica i Microbiologia (Edifici C), Universitat Autònoma de Barcelona, 08193 Bellaterra, Spain; victor.gamez@uab.es (V.G.-V.); V.Romero-Soriano@liverpool.ac.uk (V.R.-S.); joan.marti@kuleuven.be (J.M.-C.); eilasegarracarrillo@gmail.com (E.S.-C.); 2 Institute of Integrative Biology, University of Liverpool, Liverpool L69 7ZB, UK; 3 Laboratory of Clinical Virology, Department of Microbiology, Immunology and Transplantation, Rega Institute, KU Leuven, B3000 Leuven, Belgium

**Keywords:** Drosophila, piRNA pathway genes, interspecific hybrids, transposable elements, deregulation

## Abstract

Almost all eukaryotes have transposable elements (TEs) against which they have developed defense mechanisms. In the Drosophila germline, the main transposable element (TE) regulation pathway is mediated by specific Piwi-interacting small RNAs (piRNAs). Nonetheless, for unknown reasons, TEs sometimes escape cellular control during interspecific hybridization processes. Because the piRNA pathway genes are involved in piRNA biogenesis and TE control, we sequenced and characterized nine key genes from this pathway in *Drosophila buzzatii* and *Drosophila koepferae* species and studied their expression pattern in ovaries of both species and their F1 hybrids. We found that gene structure is, in general, maintained between both species and that two genes—*armitage* and *aubergine—*are under positive selection. Three genes—*krimper, methyltransferase 2,* and *zucchini*—displayed higher expression values in hybrids than both parental species, while others had RNA levels similar to the parental species with the highest expression. This suggests that the overexpression of some piRNA pathway genes can be a primary response to hybrid stress. Therefore, these results reinforce the hypothesis that TE deregulation may be due to the protein incompatibility caused by the rapid evolution of these genes, leading to a TE silencing failure, rather than to an underexpression of piRNA pathway genes.

## 1. Introduction

Transposable elements (TEs) are mobile genetic units that are interspersed throughout the genomes of almost all eukaryotes, often occupying significant fractions of the genome of their hosts. Their presence is an important threat to their host’s integrity, as their mobilising ability and repetitive nature makes them powerful endogenous mutators. To diminish their harmful effects, organisms have developed several TE repression strategies, especially in the germline, where new mutations can be transmitted to the next generations [1,2]. In the animal germline, the Piwi-interacting small RNA (piRNA) pathway acts by silencing TEs transcriptionally and post-transcriptionally through sequence homology between piRNAs and TEs [3,4,5]. piRNA biogenesis starts when long piRNA precursors are transcribed from specific genomic piRNA clusters and cleaved to produce primary piRNAs [4]. Those primary piRNAs can then be loaded into an amplification loop called the ping-pong cycle to give rise to secondary piRNAs [4,6]. Finally, transcript remnants of piRNA clusters used during secondary piRNA biogenesis are cleaved to yield new primary RNAs loaded by Piwi, which provide diversification of piRNA production [7,8]. In somatic tissues, another small-RNA mediated silencing strategy is involved in TE post-transcriptional silencing: The endogenous small interference RNA (endo-siRNA) pathway [9].

Although TEs are subject to a tight multiple-layer regulation, these strong TE repression mechanisms are sometimes overtaken under different stress conditions [10,11]. For instance, the genomic stress caused by the merge of two different genomes during interspecific hybridization can lead to the activation of endogenous TEs. TE proliferation in hybrids between species has been reported both in animals [12,13,14,15] and plants [16,17], and has been associated with a deregulation of TE expression.

The causes of TE bursts in Drosophila interspecific hybrids are still a controversial issue where different factors such as differences between maternal piRNA pools and genetic divergence between the two parental piRNA pathways come into play. Indeed, piRNA pathway genes are known to carry adaptive evolution marks [18,19] leading to cross-species incompatibilities, as observed for the piRNA pathway protein Rhino in hybrids between *Drosophila melanogaster* and *Drosophila simulans* [20]. This rapid evolution of the piRNA pathway genes was also suggested to explain their strong differences in expression between different populations of *D. simulans* [21].

Previous work in our laboratory showed that new TE insertions occur in hybrids between the species *Drosophila buzzatii* and *Drosophila koepferae* (buzzatii complex, repleta group) [15,22,23], which is likely at the origin of a genome expansion in hybrid females [24]. These TE bursts have been associated with abnormal TE expression patterns, first of the retrotransposons *Osvaldo* and *Helena* [25,26] and then in a global transcriptomic study including the whole-genome TEs [27]. Importantly, these studies proved that (i) more TE families are misregulated in F1 ovaries than in the subsequent generations of backcrossed hybrids [27], (ii) TE expression is heterogeneous between hybrid samples from different interspecific crosses [25], and (iii) there are differences in transposition rates even between hybrids of the same cross [22].

The above results could be explained by a decrease of piRNA amounts in hybrids, but our recent results showed that piRNA amounts in hybrids resemble those of the parental species with higher production [27]. This trend towards high piRNA production in hybrids suggests that the piRNA pathway might be more efficient in hybrids, which could be explained by an increase in piRNA pathway genes expression. To validate this hypothesis, we focused our study on nine piRNA pathway genes that had not been previously described in our model species. Their characterization showed that two of them, *armitage* and *aubergine*, are under positive selection. We then studied their expression in ovarian samples from individual flies, which allowed us to avoid the masking effects resulting from pooling females with different expression rates. We used qRT-PCR to evaluate the expression levels of these nine genes in F1 hybrid ovaries and localized their transcripts at a cellular level using fluorescent in situ hybridization (FISH). Our results revealed that some of the piRNA pathway genes were deregulated in the gonads of our Drosophila hybrids. This expression deregulation together with protein incompatibility—due to the rapid evolution of these genes—is likely to be related to the TE silencing failure in cross-species hybrids observed in previous studies [22,23].

## 2. Materials and Methods

### 2.1. Drosophila Stocks and Crosses

A total of four interspecific crosses (biological replicates A1, A2, B1, and B2) were performed by mating 50 *D. buzzatii* males with 50 *D. koepferae* virgin females of the same age (3 days-old) in order to obtain F1 individuals (the reciprocal cross is unsuccessful [28]). Two biological replicates, corresponding to two crosses, were analyzed for each gene: Crosses A1 and B1 were used for qRT-PCR analyses of genes *armi, aub, krimper, piwi,* and *zuc*; and crosses A2 and B2 for genes *ago3, mt2, rhino,* and *spnE*. For simplicity, they are listed as crosses A and B in the manuscript. The parental stocks used were the *D. buzzatii* Bu28 strain—an inbred line originated by the union of different populations collected in 1982 in Los Negros, Bolivia—and the *D. koepferae* Ko2 strain—an inbred line originated from a population collected in 1979 in San Luis, Argentina. Both stocks were maintained by brother–sister mating for more than a decade and are now kept by mass culturing. All stocks and crosses were reared at 25 °C in a standard Drosophila medium supplemented with yeast.

### 2.2. Sequencing of piRNA Pathway Genes in *D. buzzatii* and *D. koepferae*

Protein sequences from *D. moj*a*vensis*, *D. virilis,* and/or *D. melanogaster* associated with the nine targeted genes (Table 1) were downloaded from the Flybase database [29] and aligned to the *D. buzzatii* genome [30] using BLAST’s *tblastn*. We retrieved the best alignment hit for each gene and its *D. buzzatii* genome location was used for primer design (see Supplementary file 1A). Because no reference genome was available for *D. koepferae*, some primers did not amplify, therefore some of the genes*—aub, krimper, piwi,* and *rhino*—lack small fragments at their 5′ and/or 3′ ends. All nucleotide sequences were deposited in GenBank under the accession numbers from MN901612 to MN901629.

We carried out all PCR reactions in an MJ Research Inc. thermal cycler using the following program: 5 min at 94 °C; 30 cycles of 45 s at 94 °C, 45 s at specific annealing temperature (see Supplementary file 1A for primer sequences), 90 s at 72 °C; and 10 min at 72 °C. A final volume of 50 μL was used, with 1× High Yield Reaction Buffer with Mg2+ (Kapa Biosystems), 0.2 mM of each dNTP (Roche), 0.4 μM of each primer (Sigma-Aldrich), template DNA (10–20 ng) and 0.04 U/μL of Taq polymerase (KapaTaq from Kapa Biosystems). S*pnE* and *aub* genes were amplified using Roche’s Expand Long Template PCR system (for both parental species). Amplicons were purified with the Nucleospin Gel and PCR Clean-Up kit (Macherey-Nagel), and cloned with the pGEM-T Easy Vector System I (Promega).

### 2.3. Sequence Analysis

Sequencing of PCR cloned products was performed by Macrogen Inc. (Seoul, Korea) service. Multiple sequence alignment was carried out with MAFFT [31]. For transcript prediction and consensus protein domain motifs finding, ORF Finder (https://www.ncbi.nlm.nih.gov/orffinder/) and Conserved Domain Search [32] tools were used respectively. The Augustus sofware [33] was used for gene structure prediction in silico. Those predicted genes were compared to existing annotations in *D. mojavensis* and *D. melanogaster* species (data obtained from FlyBase database: http.//www.flybase.org, January 2019). TE intron insertions were detected using Repeat Masker software [34] in the species under study and *D. mojavensis* (see Supplementary file 3C). To test signatures of selection we performed McDonald and Kreitman test (MK) using DnaSP v6 software [35]. The intraspecific polymorphism was computed by aligning the piRNA pathway genes from the *D. buzzatii* line considered in this study, to those of the previous sequenced *D. buzzatii* genome [30] and then compared to *D. koepferae* sequences.

### 2.4. Quantification of Gene Transcripts by qRT-PCR

Ovaries of 5- or 6-day-old flies (from parental species or F1 hybrids) were dissected in PBT (1× phosphate-buffered saline [PBS], 0.2% Tween 20). Total RNA was purified individually for each fly’s pair of ovaries with the Nucleospin RNA purification kit (Macherey-Nagel). cDNA synthesis was carried out with anchored-oligo(dT)18 primers using Roche’s Transcriptor First Strand cDNA Synthesis Kit. Transcript abundance was estimated by fluorescence intensity using Biorad’s iQ SYBR Green Supermix on a CFX96 BioRad Real-Time lightcycler. We performed relative quantification using the ribosomal *rp49* housekeeping gene as endogenous control, with at least two technical replicates per sample. This control gene showed to be equally expressed in *D.buzzatii* and *D. koepferae* ovaries in a previous work using the same primers and stocks [25].

For each gene we used the same primer set in both species (Supplementary file 1B), designed in a conserved region and tested to have similar efficiencies in *D. buzzatii* and *D. koepferae* (Supplementary file 2A). Thus, expression rates were calculated using the comparative Ct method [36] as in [25] (supplementary file 2B). For each gene, we analyzed five sample groups: 2 maternal *D. koepferae* groups (crosses A and B), 2 F1 hybrid groups (female offspring from crosses A or B) and one *D. buzzatii* group (females of the stock, not involved in the cross).

### 2.5. Fluorescent In Situ Hybridization in Ovaries

Ovaries of 3-days old flies (which is the ideal age for optimal visualization of the different cells from ovaries) were dissected in PBT, following the protocol described in [37]. Antisense RNA probes for the 9 genes (see Supplementary file 4 for probes details) of the piRNA pathway, including T7 and SP6 promoter sites, were labeled by in vitro transcription of SP6/T7 using the DIG RNA Labeling Kit (Roche) and used to detect gene expression in ovaries. Hybridization signal was detected using the anti-DIG POD antibody (Roche) and fluorescence amplification (TSA PLUS Cyanine3 kit, PerkinElmer), and visualized with an Olympus Fluoview 1000 confocal scanning laser microscope.

### 2.6. Statistical Methods

We used IBM SPSS 22 software for statistical analyses. As the assumptions of Gaussian distribution and equal variances are not valid in qRT-PCR experiments with small sample sizes, we used the non-parametric Wilcoxon rank sum test (or Mann–Whitney test [38]) to compare expression rates between hybrids and parental species. Kruskal–Wallis test [39] was used to determine whether differences between all groups were significant. All multiple test corrections were achieved using a False Discovery Rate (FDR) threshold of 5% based on the method of Benjamini-Hochberg [40]. Additionally, Levene’s test for equality of variances, was used to assess changes in variance between groups.

## 3. Results

### 3.1. piRNA Pathway Gene Characterization in *D. buzzatii* and *D. koepferae*

In order to perform gene characterization and expression analyses we sequenced nine piRNA pathway genes in our parental species, *D. buzzatii* and *D. koepferae*. These genes have never been characterized in these species before—they are not annotated in the available *D. buzzatii* genome sequence [30], and no genome sequence has been released to date for *D. koepferae*. Our multiple sequence alignments (MSA) show that *ago3* is the most conserved gene between both species with 95.6% of nucleotide identity in the coding sequence and rhino is the most divergent with 78% of identity (Table 1). We observe that although amino acid similarities between parental species highly differ between genes (ranging from 69% for *rhino* to 95.6% for piwi), gene structure is generally conserved. Indeed, the number of exons is exactly the same between *D. buzzatii* and *D. koepferae*, and does not change when compared to their closest sequenced relative, *D. mojavensis* (Supplementary file 3A), or even to the more distant species *D. melanogaster* (Supplementary file 3B).

In the case of *ago3*, the first intron is seven times larger in *D. koepferae* (2275 bp) than in *D. buzzatii* (321bp), likely due to transposition events. In fact, fragmented TE sequences represent 42% of the *D. koepferae* first intron length (including both retrotransposons and DNA transposons, see Supplementary file 3C). Even though the same intron in *D. buzzatii* does not carry any TE sequence, these are also present in the orthologous sequence of *D. mojavensis*, the closest species with a sequenced genome. Interestingly, this gene sequence is the most conserved between our parental species (Table 1).

We performed the McDonald and Kreitman (MK) test [41] to test for putative selection marks in the nine studied genes (Table 2). The proportion of adaptive substitutions (α) is higher than 0 for all genes, indicating that they are likely under selective pressure, although only *armi* and the region corresponding to the PAZ domain of *aub* yield significant results.

### 3.2. Gene Expression in Parental Species

Gene expression in parental species ovaries was studied in individual flies using a single pair of ovaries per sample. mRNAs were quantified by quantitative real time PCR (qRT-PCR) using the comparative C_T_ method [36]. In order to achieve higher statistical power, five groups were used for measuring and comparing gene expression: One parental *D. buzzatii* group (Dbu, not involved in the cross), two *D. koepferae* maternal groups (DkoA and DkoB), used subsequently to obtain the two respective hybrid offspring groups (HybA and HybB).

**Figure 1:**
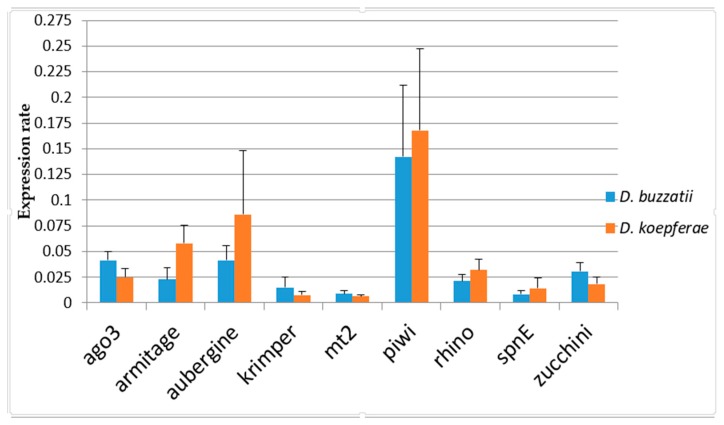
Expression rates in the parental species *D. koepferae* and *D. buzzatii*. Note that *D. buzzatii* females are not involved in the cross. For *D. koepferae* samples the mean between two families involved in the crosses is shown. Error bars represent standard deviation.

Expression differences between parental species (Figure 1 and Table 3) are statistically significant for all the studied genes except for *piwi* (*p* = 0.428). *Aub* shows the largest ER difference between parental species (2.24-fold difference, ER_Dbu_ = 4.12 × 10^−2^, ER_Dko_ = 8.62 × 10^−2^). However, these differences do not follow a single trend, since some genes are more expressed in *D. koepferae* (*armi, aub, piwi,* and *snpE*) while others are more expressed in *D. buzzatii* (*ago3, krimp,* and *zuc*).

### 3.3. Gene Expression in Hybrids

**Figure 2:**
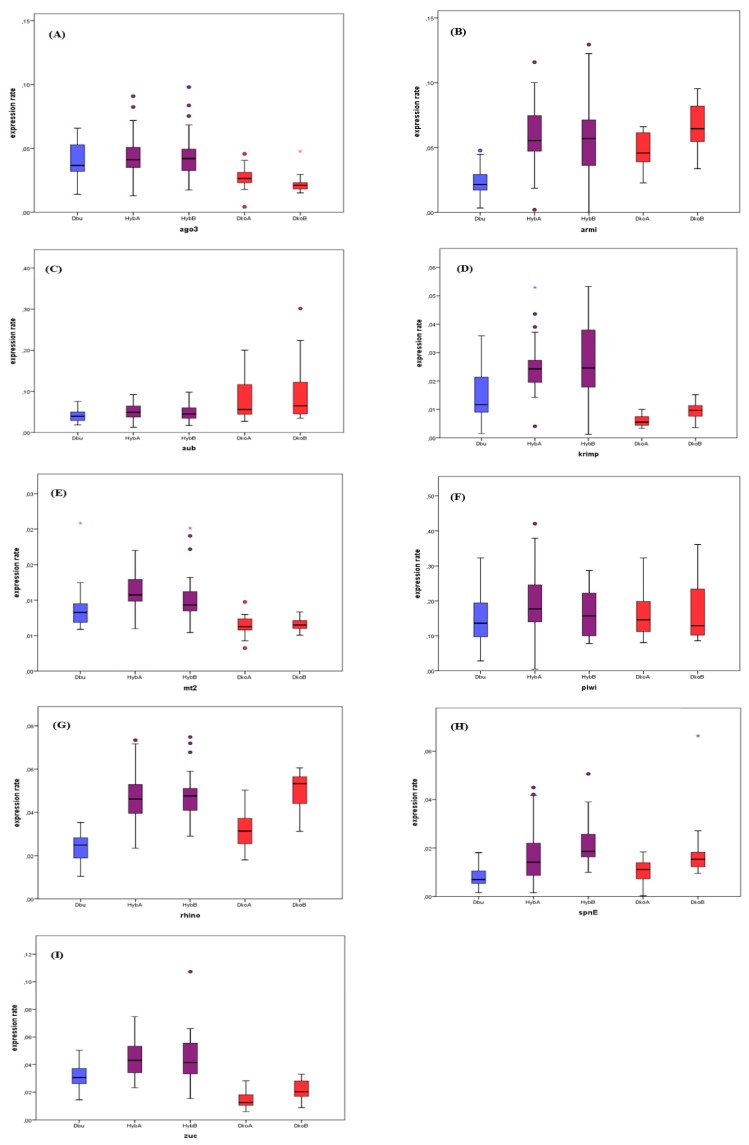
Expression rates relative to *rp49* housekeeping gene in parental species (*Dko* and *Dbu*) and hybrids. Boxes are determined by the first and third quartile values, with an intermediate deep line corresponding to the median value. Circles correspond to outliers (above or below 1.5-fold the interquartile range) and asterisks correspond to atypical values. Every group is shown in the same order in every plot: *Dbu* parental species, hybrids groups A and B and *Dko* maternal species groups A and B. Graphics from (**A**) to (**I**) refer to each studied gene.

We quantified the expression of the same nine piRNA pathway genes in ovaries of hybrid females as previously described for parental species (Figure 2). The ERs values were calculated in hybrids obtained in two different crosses (HybA and HybB, see Methods) and compared to their respective maternal group (DkoA or DkoB) as well as to *D. buzzatii* (Dbu, females were not involved in the cross). We tested for differences in ER within groups (Dbu, DkoA, DkoB, HybA, and HybB) for the nine studied piRNA pathway genes using the Kruskal-Wallis test, and found significant results for all of them, except for *piwi* (see Supplementary file 5).

We then performed one-to-one comparisons between groups using the Wilcoxon sum rank test (Table 4). The analyses revealed three possible scenarios: (a) ERs were not significantly different between hybrids and parental species—no difference scenario, (b) ERs were significantly higher in hybrids than in one of the parental species—Dbu or Dko-biased expression scenario, or (c) ERs were higher in hybrids than in both parental species—hybrid overexpression scenario (see Table 4 and Figure 2). A single gene, *piwi,* did not show any significant difference between hybrids and parents (Table 4 and Figure 2F). *Ago3* had a Dbu-biased expression (higher than *D. koepferae*, Figure 2A and Table 4), while *rhino,* s*pnE,* and *armi* presented Dko-biased expression (higher than *D. buzzatii*, Figure 2B,G,H and Table 4). A total of three genes—*krimp*, *mt2,* and *zuc*—fell in the hybrid overexpression scenario (Figure 2D,E,I and Table 4). In the case of *aub*, cross A displayed no significant differences between hybrids and parental species, whereas in cross B the hybrid expression is significantly different to the most expressed parental species, Dko (Figure 2A and Table 4). Moreover, this significance in cross B is still maintained after removing the outlier point (Figure 2A) W = 177 and *p* = 0.013 * (data not shown).

Studying individual fly samples allowed us to test for differences in variability between groups. Using Levene’s test for equality of variances, we showed that all genes had significant differences in variance within groups except *piwi* and *rhino* (see Supplementary file 6A). Only z*uc* and *spnE* genes showed higher variance values in hybrids than in both parental species (Supplementary file 6B). Indeed, they presented high ER individual variability in hybrids: they were overexpressed in some individuals and underexpressed in others, when compared to the parental median value (Figure 2).

### 3.4. Expression Localization Patterns in Hybrid and Parental Species Ovaries

To assess whether the observed quantitative differences in gene expression between hybrids and parents involved changes in the localization of the transcripts, we performed fluorescent in situ hybridization (FISH) in ovarian tissue in order to detect the mRNA location of the genes under study.

**Figure 3:**
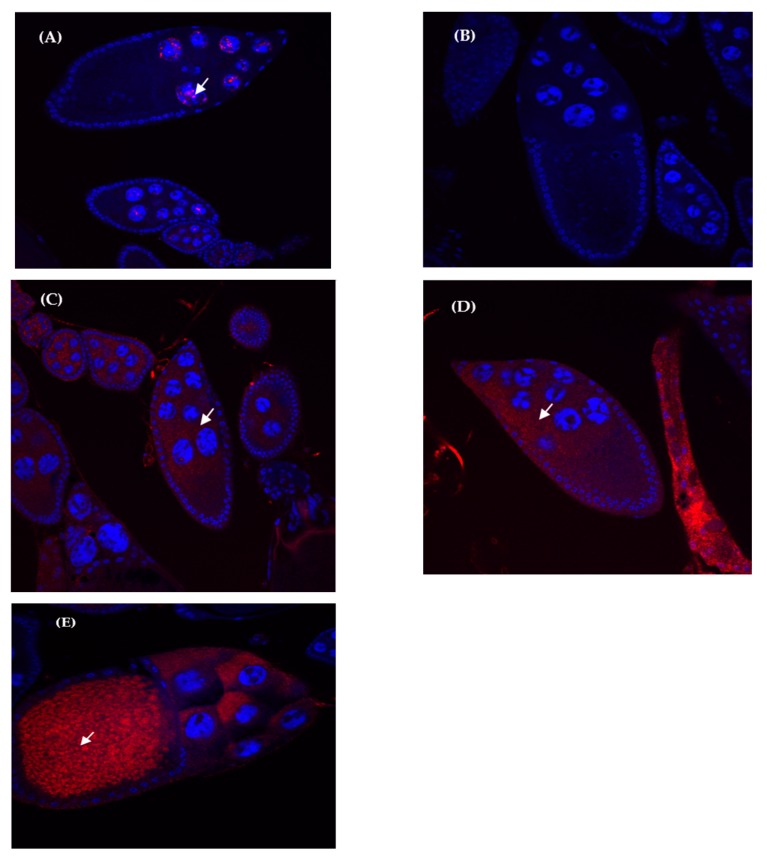
Fluorescent in situ hybridization (FISH) of *ago3* RNA expression in ovaries. Red staining are *ago3* transcripts, blue staining is DAPI (cells nuclei). Arrows mark the presence of *ago3* transcripts. (**A**) positive control using *Osvaldo* retrotransposon probe, (**B**) negative control, (**C**) *D. buzzatii*, (**D**) *D. koepferae*, (**E**) hybrid.

All genes showed expression mainly in the cytoplasm of nurse cells (see Supplementary file 7), both in parental species and hybrid ovaries. A faint expression signal can also be detected inside the nucleus of nurse cells in some cases, likely corresponding to recently transcribed mRNAs. Interestingly, transcript location for *ago3* showed a different pattern between hybrids (Figure 3E) and parents (Figure 3C,D): a clear and strong hybridization signal was detected in the hybrid oocytes, whereas only faint signals were detected in parental species. Additionally, we found some cases in which signal intensity seems to follow the same trend as in qRT-PCR—for instance, for *mt2* the expression is higher in hybrids than in parental species. However, this can only be used as a validation since FISH is not a quantitative technique.

## 4. Discussion

### 4.1. piRNA Pathway Gene Structure is Conserved between *D. buzzatii* and *D. koepferae*

Nine piRNA pathway genes were sequenced for the first time in the parental species *D. buzzatii* and *D. koepferae*. Four of them have an exon number higher than the average in *D. buzzatii* genome (3.8 exons, [30]). All genes but *ago3* have an identical number of exons/introns in both parental species as well as in *D. melanogaste*r and *D. mojavensis*, which was expected given that 80% of intron positions are conserved across distant eukaryotes [42]. *Armi* and *aub* bear marks of positive selection (Table 2), in concordance with a previous study of RNA interference genes across the Drosophila phylogeny [43].

Although the general gene structure of the studied piRNA pathway genes is conserved among Drosophila species, *ago3* caught our attention because of its low exon/intron number in our species compared to *D. melanogaster* (2 vs. 6 exons respectively). *Ago3* has a highly variable exon number within the Drosophila genus, from a single exon in *D. suzukii* and *D. pseudoobscura* to eight exons in *D. virilis* [43]. This variability cannot be explained phylogenetically, as *ago3* extreme exon numbers (high and low) occur in species of both the Drosophila and the Sophophora subgenus. Hence, we cannot be sure whether this variability is due to intron gain or intron loss processes. Although intron loss is predominant over intron gain in Drosophila [44], the presence of TE sequences in the species with intron-rich *ago3* indicates that transposition-driven intron gain might have occurred [45]. Indeed, the predominance of intron gain has been attributed to selective pressures due to large effective population sizes [44], which would not explain a lower intron number in our species, whose population sizes are lower than in *D. melanogaster* [46,47].

### 4.2. Armitage and Aubergine Bear Marks of Positive Selection

The nine piRNA pathway sequenced genes in this study showed identity values between *D. buzzatii* and *D. koepferae* ranging between 78–95.6% for DNA and 69–95.6% for protein sequences, a rather low degree of conservation for a couple of species that diverged approximately 5 Mya [48]. This suggests that piRNA pathway genes tend to evolve quickly compared to other genes, as observed in multiple invertebrates [49] and in a previous work [27] where these genes showed protein identity values lower than the median of the proteome between our parental species. Despite the low number of sequences analyzed, we found that at least two of these genes (*armi* and *aub*) are under positive selection in our model species (Table 2), which is in concordance with previous studies showing that piRNA pathway display high rates of adaptive evolution [19,20,42]. It is important to note that in *aub* these selection marks were only detected in the PAZ protein domain, whereas the whole gene is affected in *armi*. Because some domains are shared by different piRNA pathway genes (e.g., the PAZ domain), and positive selection marks were not observed in all of them, we deduced that adaptation could be gene-specific rather than domain-specific. Several studies have suggested that the degree of gene adaptive evolution is correlated with the position of the corresponding protein in the interaction network [42,50]. In the piRNA pathway, the fastest evolving components of piRNA pathway do not usually correspond to effector proteins [51]. In our case this is true for *ago3* and *piwi* (that are effector proteins with no significant positive selection marks) but not for *aub*, which shows a greater effect of positive selection in its PAZ domain. In concordance with our results, *armi* has a general trend to show positive selection marks in different and independent tests [52]. Signatures of adaptation are a pervasive effect in genes affecting piRNA synthesis, although this high evolution rate is not only restricted to this pathway: most of the genes related to RNA interference pathways have also been reported to display high rates of adaptative evolution [49]. Besides, as these genes participate in TE silencing, it is important to take into account the evolutionary process of host–pathogen interaction or “Red Queen” host–pathogen arms race [52]. This rapid evolution of the piRNA pathway genes is a key process in species divergence and can easily generate orthologous incompatibility after hybridization barrier [20].

### 4.3. Misexpression of piRNA Pathway Genes in *D. buzzatii–D. koepferae* Hybrids

The combination of divergent Drosophila genomes during hybridization results in a genomic shock characterized, inter alia, by a TE deregulation [22,23,25,26] caused by a failure of TE silencing [20,27,53]. There is limited information linking TE deregulation with expression failures in piRNA pathway genes [27], well-known by their important role in germinal TE regulation. Our study has quantified the individual expression, in ovaries, of nine key piRNA pathway genes in *D. buzzatii*, *D. koepferae* and their F1 hybrids. We observed that the median expression values in hybrids tend to be higher than at least one parental species in all genes except *aub*, which codes for an effector protein (Figure 2). However, hybrid expression values were only significantly higher than in both parents for *krimp*, *mt2,* and *zuc*. For the four other genes (*ago3, rhino, spnE,* and *armi*), hybrid expression was only significantly higher than the parental species with the lowest expression. Finally, *piwi* expression was not significantly different between hybrids and parents, while *aub* presented different results between crosses. These results do not completely match the previous RNA-seq study in the same species, in which most genes had expression levels similar to the parental species with the highest expression [27]. These differences likely lie in the fact that here we analyzed each ovary pair individually whereas the previous study pools the ovaries resulting from different hybrid crosses. It is worth highlighting that different individuals (both within parents and hybrids) also showed a high variability of piRNA pathway gene expression reaching differences of up to 2.5-fold.

*zuc* and *spnE* are the only genes that displayed higher inter-individual variability in hybrids compared to both parental species (see Supplementary file 6). However, because hybrids are known by their high genome instability, these differences could be due to stochastic genetic and epigenetic changes that do not involve the meiotic process, as suggested by other authors [54]. These results are in concordance with previous studies in hybrids showing high individual and cross heterogeneity in transposition [22] and expression rates [25] of the retrotransposon *Osvaldo*. In the same way, expression studies on the retrotransposon *Helena* showed additive patterns of expression in hybrids compared to parental species when using pooled flies in qRT-PCR experiments, while FISH experiments in individual flies showed a more extensive presence of *Helena* transcripts in F1 hybrids compared to parental species [26]. In the present study, the transcripts of the studied genes were mainly localized in the ovarian nurse cells’ cytoplasm in both hybrids and parental species. For *Ago3*, a strong transcript signal was also detected in the oocyte cytoplasm of hybrid ovaries, while only a faint signal was detected in parental ones. It is known that ovarian nurse cells transfer mRNAs and proteins into the oocyte for the production of the egg and early embryo [55]. However, *ago3* is the only gene that seems to be more expressed in the oocyte of hybrids than of parental species, which might be due to an activation of the piRNA pathway to counteract TE deregulation in hybrids, or to an abnormal localization of the transcripts due to hybrid incompatibilities. Indeed, abnormal distributions of tissue expression were previously reported in Drosophila hybrids [26].

Several studies showed that interspecific hybrids tend to present TE derepression compared to parental species [53,56,57,58,59]. However, repression cases have also been observed for some TEs, pointing out a more complex alteration of the TE regulation network. Our results show that nine piRNA pathway genes have a non-uniform expression pattern between hybrids, and that three genes—*krimp*, *mt2,* and *zuc*—are overexpressed in hybrids compared to parental species. Intuitively, we could think that hybrid TE derepression might be preceded by the underexpression of regulatory genes. However, the overexpression of some piRNA pathway genes could be a genomic response to the stress caused by TE mobilization during interspecific hybridization events, to counteract harmful effects on the cell. Indeed, although a reduction of the ping-pong cycle efficiency seems to occur in hybrids for *Helena*-specific piRNAs [26], the general trend for whole-genome TEs [27] is to show additive or higher ping-pong signature levels in hybrids than in parental species. In the same way, non-deficient amounts of total piRNA were observed also observed in our previous studies [27].

All in all, the most plausible hypothesis to explain TE deregulation in *D. buzzatii-koepferae* hybrids is the functional divergence between parental piRNA pathways, especially in terms of piRNA production efficiency. Indeed, here we show that some piRNA pathway genes evolve under positive selection and show lower conservation than expected in species that diverged 4.5 Mya. These results are in agreement with our previous transcriptomics study, in which we showed that most piRNA pathway proteins (predicted in silico) have identity percentages between *D. buzzatii* and *D. koepferae* lower than the median of the whole proteome [27]. The accumulated divergence between piRNA pathway proteins has also been proposed to explain TE deregulation in *D. melanogaster-D. simulans* hybrids [53] and was recently attributed to the lack of Rhino and Deadlock protein binding in hybrids [20].

Still, further research is needed for a better understanding of TE deregulation in interspecific hybrids, including studying how the amount of effector proteins affects the piRNA pathway breakdown, as well as whether and how epigenetic changes (such as histone methylation) are involved in TE deregulation.

## 5. Conclusions

Genomic stress caused by interspecific hybridization, induces TEs misregulation in Drosophila where piRNA pathway genes can play an important role. In this study, we characterized and quantified the expression of nine piRNA pathway genes in *D. koepferae* and *D. buzzatii* species, together with their interspecific hybrids. We showed that at least two of these genes (*armi* and *aub*) are under adaptive selection, despite being closely related species. Hybrid ovaries showed deregulation of some piRNA pathway genes compared to parental species and a trend to the overexpression in *krimp*, *mt2,* and *zuc*. This result, together with the observation of a non-deficient amount of piRNAs in hybrids in previous studies, reinforces the idea that the overexpression is a cellular response to mitigate hybrid stress. Therefore, the TE deregulation in hybrids might be due, at least in part, to protein incompatibility due to the rapid evolution of some of the genes under selective pressure such as *armi* and *aub*.

## Figures and Tables

**Table 1:** Structure of the piRNA pathway genes sequenced region.

	*D. buzzatii*				*D. koepferae*					
Genes ^a^	Length	CDS	Protein	Exons	Length	CDS	Protein	Exons	NI (%)	PS (%)
*ago3*	2009	1688	562	2	3961	1686	562	2	95.6	93.2
*armi*	3701	3432	1141	5	3688	3424	1122	5	93.2	92.5
*aub*	4250	2562	852	9	4474	2466	821	8^b^	94	92.9
*krimp*	1929	1852	617	2	1920	1843	615	2	92.5	90.9
*mt2*	812	810	270	1 ^b^	1064	1005	335	2	94	94.4
*piwi*	3924	2625	874	8	2484	2097	699	7^b^	94.5	95.6
*rhino*	1865	1749	602	2	1944	1885	628	2	78	69
*spnE*	6052	4012	1337	11	6245	4125	1375	11	94	91.1
*zuc*	651	651	217	1	654	654	218	1	86.5	72.7

This table contains the main features of the nine studied genes, including the length of their sequences, of their transcripts, and of their coding sequence (CDS) in base pairs (bp); the length of the translated protein in amino acids, and the number of exons of each transcript. Identity percentages were calculated using BLAST alignments between *D. koepferae* and *D. buzzatii* coding sequences and translated protein sequences. NI: Nucleotide identity; PS: Protein similarity; ^a^: *ago3: argonaute 3, armi: armitage, aub: aubergine, krimp: krimper, mt2: methyltransferase 2, spnE: spindle E, zuc: zucchini*. ^b^: lower number of exons due to incomplete sequencing.

**Table 2:** Results of McDonald and Kreitman (MK) test comparing *D. buzzatii* and *D. koepferae* sequences.

Genes	Region/Domain	Pn/Ps	Dn/Ds	NI	α	*P*
*ago3*	CDS	-	0.911	-	-	-
*armi*	CDS	8.33 × 10^−2^	6.35 × 10^-1^	1.31 × 10^-1^	8.69 × 10^-1^	1.00 × 10^-3 **^
*aub*	CDS	5.00 × 10^-1^	6.37 × 10^-1^	7.84 × 10^-1^	3.27 × 10^-1^	2.15 × 10^-1^
*krimp*	CDS	0.00	8.25 × 10^-1^	0.00	1.00	6.00 × 10^-2^
*mt2*	CDS	1.67 × 10^-1^	4.38 × 10^-1^	3.80 × 10^-1^	6.19 × 10^-1^	3.75 × 10^-1^
*piwi*	CDS	3.08 × 10^-1^	4.33 × 10^-1^	7.11 × 10^-1^	2.88 × 10^-1^	5.78 × 10^-1^
*rhino*	CDS	7.50 × 10^-1^	4.94 × 10^-1^	6.58 × 10^-1^	3.41 × 10^-1^	5.89 × 10^-1^
*spnE*	CDS	5.00 × 10^-1^	9.31 × 10^-1^	4.65 × 10^-1^	5.34 × 10^-1^	1.65 × 10^-1^
*zuc*	CDS	5.00 × 10^-1^	3.34 × 10^-1^	6.67 × 10^-1^	3.32 × 10^-1^	7.44 × 10^-1^
*aub*	PAZ	1.60	6.30 × 10^-1^	9.94 × 10^-1^	5.00 × 10^-3^	7.5 × 10^-1^ ***

CDS: coding sequence; Pn/Ps: polymorphic changes and Dn/Ds: divergent changes—s refers to neutral sites and n to non-neutral ones. NI: Neutrality Index ((Pn/Ps)/(Dn/Ds)); α: proportion of adaptive substitutions (1-NI); p: *p*-value after Jukes–Cantor correction. **: *p* < 0.01; ***: *p* < 0.001. MK test was performed for the complete CDS (results for all genes shown) and for each individual domain (only domains with significant results are shown). PAZ: protein binding domain found in Piwi, Argonaute, and Zwille proteins. MK test could not be performed for *ago 3* due to the low gene polymorphism.

**Table 3:** Comparison between *D. koepferae* and *D. buzzatii* expression levels using the Wilcoxon Rank sum test.

	*D. koepferae* vs. *D. buzzatii*	
Gene	W	*p*-Value
*ago3*	790	1.46 × 10^-6^ ***
*armi*	36	1.24 × 10^-7^ ***
*aub*	190	1.31 × 10^-3^ **
*krimp*	582	4.98 × 10^-3^ **
*mt2*	773	9.00 × 10^-5^ ***
*piwi*	349	4.28 × 10^-1^
*rhino*	118	3.00 × 10^-6^ ***
*spnE*	232	9.21 × 10^-4^ ***
*zuc*	691	1.25 × 10^-5^ ***

W = Wilcoxon Rank sum test statistic. **: *p* < 0.01; ***: *p* <0.001. All *p*-values were corrected using a False Discovery Rate threshold of 5%.

**Table 4:** Comparison of the different gene expression levels between hybrids and parental species (*D. koepferae* and *D. buzzatii*).

Gene	Cross	N	Median	SD	vs*. D. buzzatii*		vs. *D. koepferae* A		vs. *D. koepferae* B	
					W	*p*-Value	W	*p*-Value	W	*p*-Value
*ago3*	**A**	45	4.11 × 10^-2^	1.28 × 10^-2^	143	8.41 × 10^-1^	31	1.02 × 10^-2^ *		
	**B**	26	4.20 × 10^-2^	1.88 × 10^-2^	136	7.51 × 10^-1^			0	0.00 ***
*armi*	**A**	36	5.53 × 10^-2^	2.33 × 10^-2^	207	0.00 ***	152	1.05 × 10^-1^		
	**B**	34	5.69 × 10^-2^	2.74 × 10^-2^	210	0.00 ***			156	6.20 × 10^-2^
*aub*	**A**	36	4.90 × 10^-2^	1.87 × 10^-2^	75	4.67 × 10^-1^	52	7.20 × 10^-2^		
	**B**	34	4.49 × 10^-2^	2.05 × 10^-2^	90	6.90 × 10^-1^			32	9.00 x 10^-3^ **
*krimp*	**A**	36	2.43 × 10^-2^	8.80 × 10^-3^	38	2.00 × 10^-2^ *	0	0.00 ***		
	**B**	34	2.46 × 10^-2^	1.27 × 10^-2^	37	1.80 × 10^-2^ *			1	0.00 ***
*mt2*	**A**	45	1.07 × 10^-2^	2.30 × 10^-3^	58	1.80 × 10^-2^ *	1	0.00 ***		
	**B**	26	9.32 × 10^-3^	4.50 × 10^-3^	86	1.01 × 10^-1^			1	0.00 ***
*piwi*	**A**	36	1.77 × 10^-1^	8.81 × 10^-2^	66	1.81 × 10^-1^	91	6.01 × 10^-1^		
	**B**	34	1.57 × 10^-1^	6.85 × 10^-2^	102	9.11 × 10^-1^			84	4.33 × 10^-1^
*rhino*	**A**	45	1.31 × 10^-2^	1.01 × 10^-2^	246	1.70 × 10^-2^ *	149	1.09 × 10^-1^		
	**B**	26	1.86 × 10^-2^	9.00 × 10^-3^	295	0.00 ***			156	6.20 × 10^-2^
*spnE*	**A**	45	1.31 × 10^-2^	1.01 × 10^-2^	55	1.70 × 10^-2^ *	61	1.09 × 10^-1^		
	**B**	26	1.85 × 10^-2^	9.00 × 10^-3^	5	0.00 ***			54	6.20 × 10^-2^
*zuc*	**A**	36	4.30 × 10^-2^	1.31 × 10^-2^	48	4.70 × 10^-2^ *	1	0.00 ***		
	**B**	34	4.14 × 10^-2^	1.78 × 10^-2^	59	1.96 × 10^-1^			12	2.00 × 10^-3^ **

N = number of samples analyzed; SD = Standard Deviation; W = Wilcoxon Rank sum test statistic. *: *p* < 0.05; **: *p* < 0.01; ***: *p* < 0.001. All showed *p*-values were corrected using False Discovery Rate.

## Supplementary Materials

The following are available online at https://www.mdpi.com/2073-4425/11/2/215/s1, Supplementary file 1: A: primers used for each gene sequencing. B: primers used for qRT-PCR; Supplementary file 2: A: Primer efficiencies. B: qRT-PCR raw data; Supplementary file 3: A: piRNA pathway genes features in *D. mojavensis*, the closest relative to *D. buzzatii* and *D. koepferae* with a sequenced genome. B: piRNA pathway genes main feature in the genetic model *D. melanogaster*; Supplementary file 4: primers used for FISH probes, including probes length and the exon they are in Supplementary file 5: Comparison of the different gene expression levels between parental species (*D. koepferae* and *D. buzzatii*) and hybrids using Kruskal–Wallis test. Supplementary file 6: A: Levene test for homogeneity of variances. All p-values are corrected using the False Discovery Rate test. B: Levene test for homogeneity of variances between hybrids and parental species. All p-values are corrected using the False Discovery Rate test Supplementary file 7: FISH pictures showing mRNA localization for analyzed genes in ovaries. Red staining are gene transcripts, blue staining is DAPI (cells nuclei). Arrows mark the presence of gene transcripts.
