# Supplementary material for: Drosophila Interspecific Hybridization Causes a Deregulation of the piRNA Pathway Genes
Source: Genes (Basel). 2020 Feb 19;11(2):215. doi: 10.3390/genes11020215 (PMC7073935; doi:10.3390/genes11020215)
Supplement: Supplementary file 1 [file genes-11-00215-s001.zip › supplementary/Supplementary file 7.docx]

**Additional file 7:**
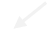

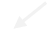
FISH pictures showing mRNA localization for analyzed genes in ovaries. Red staining are gene transcripts, blue staining is DAPI (cells nuclei). Arrows mark the presence of gene transcripts.

***armitage***

***
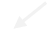

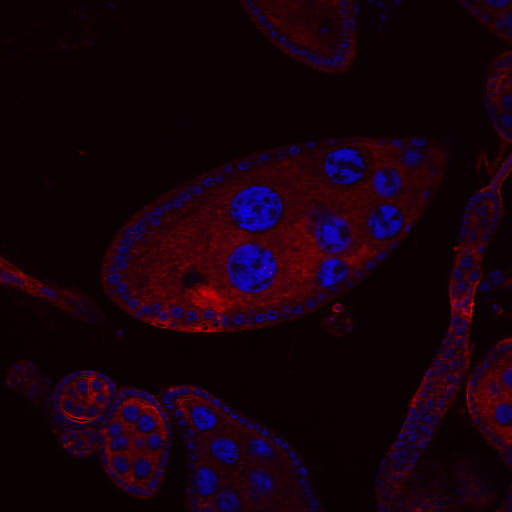

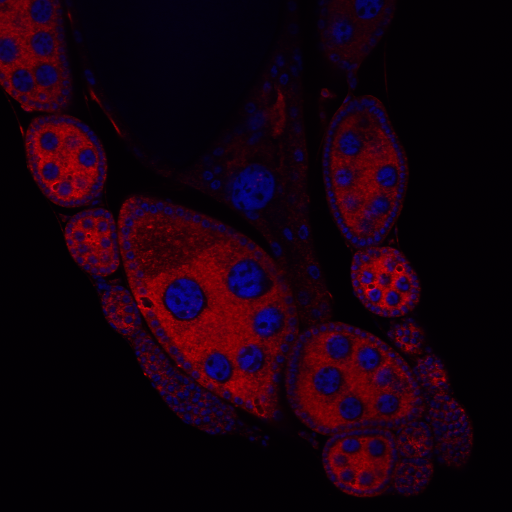
D. buzzatii D. koepferae***

***
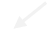

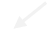

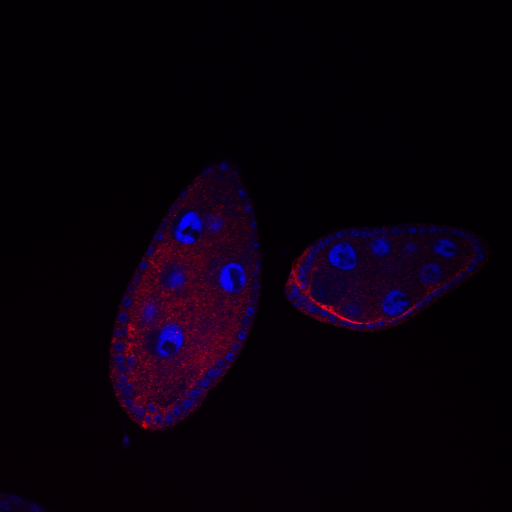
***

**Hybrids**

***aubergine***

***
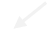

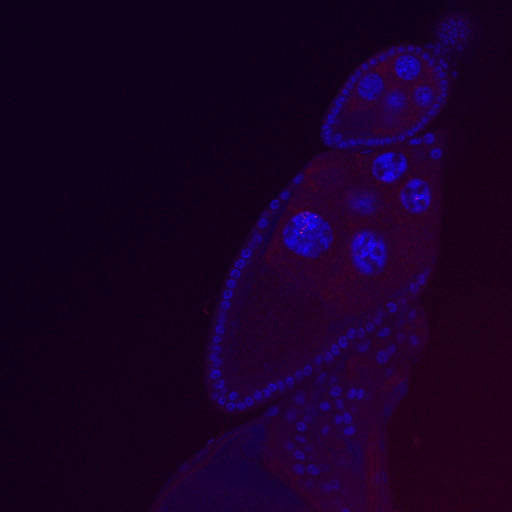

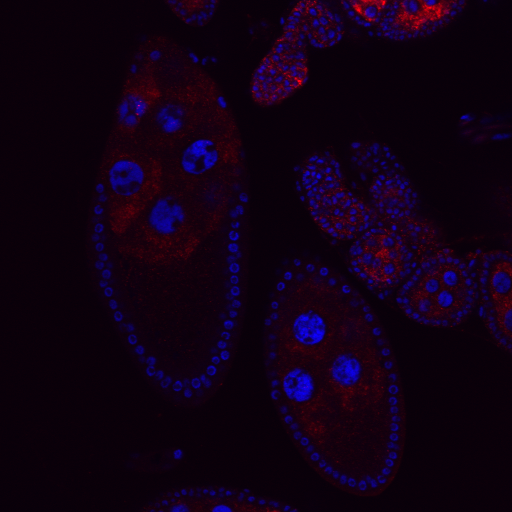

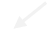
D. buzzatii D. koepferae***

**Hybrids**

***
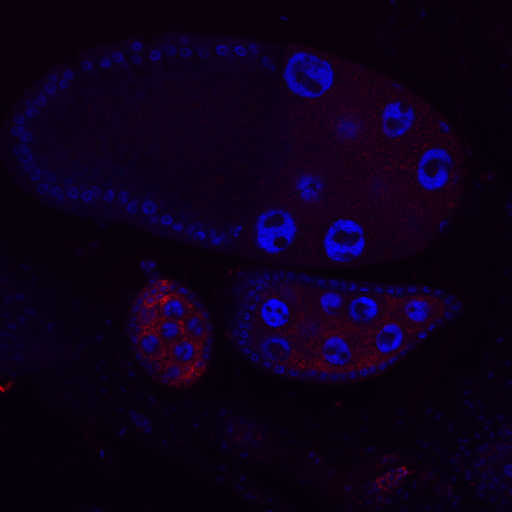
***

***
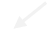
***

***krimper***

***D. buzzatii D. koepfeare***

***
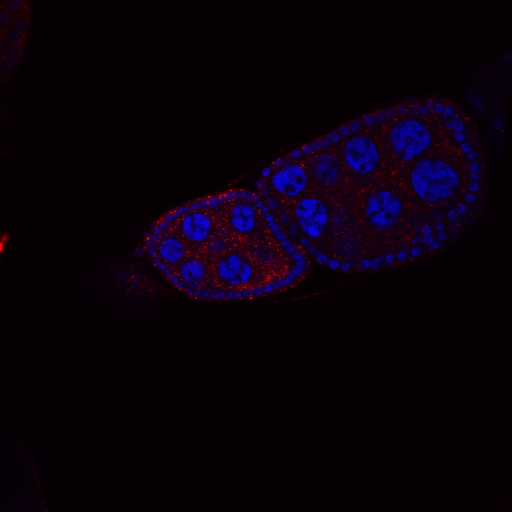

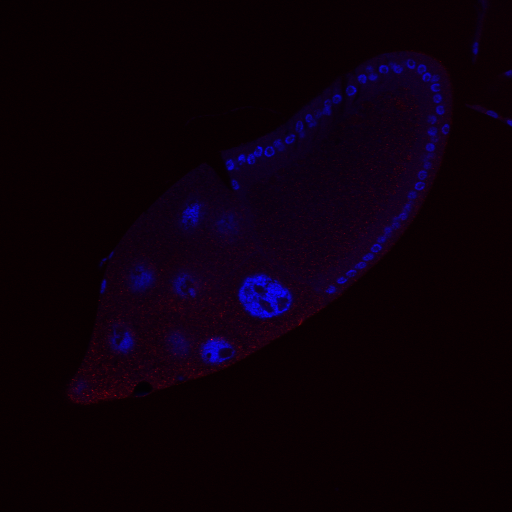
***

***
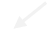

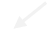
***


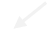


**Hybrids**

***
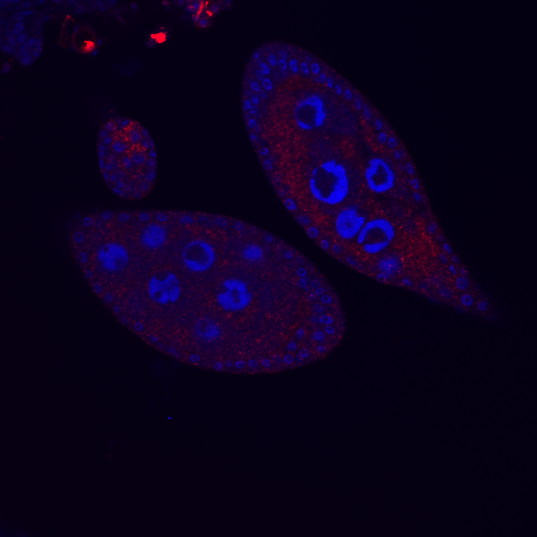
***

***metiltransferase***

***D. buzzatii D. koepfeare***

***
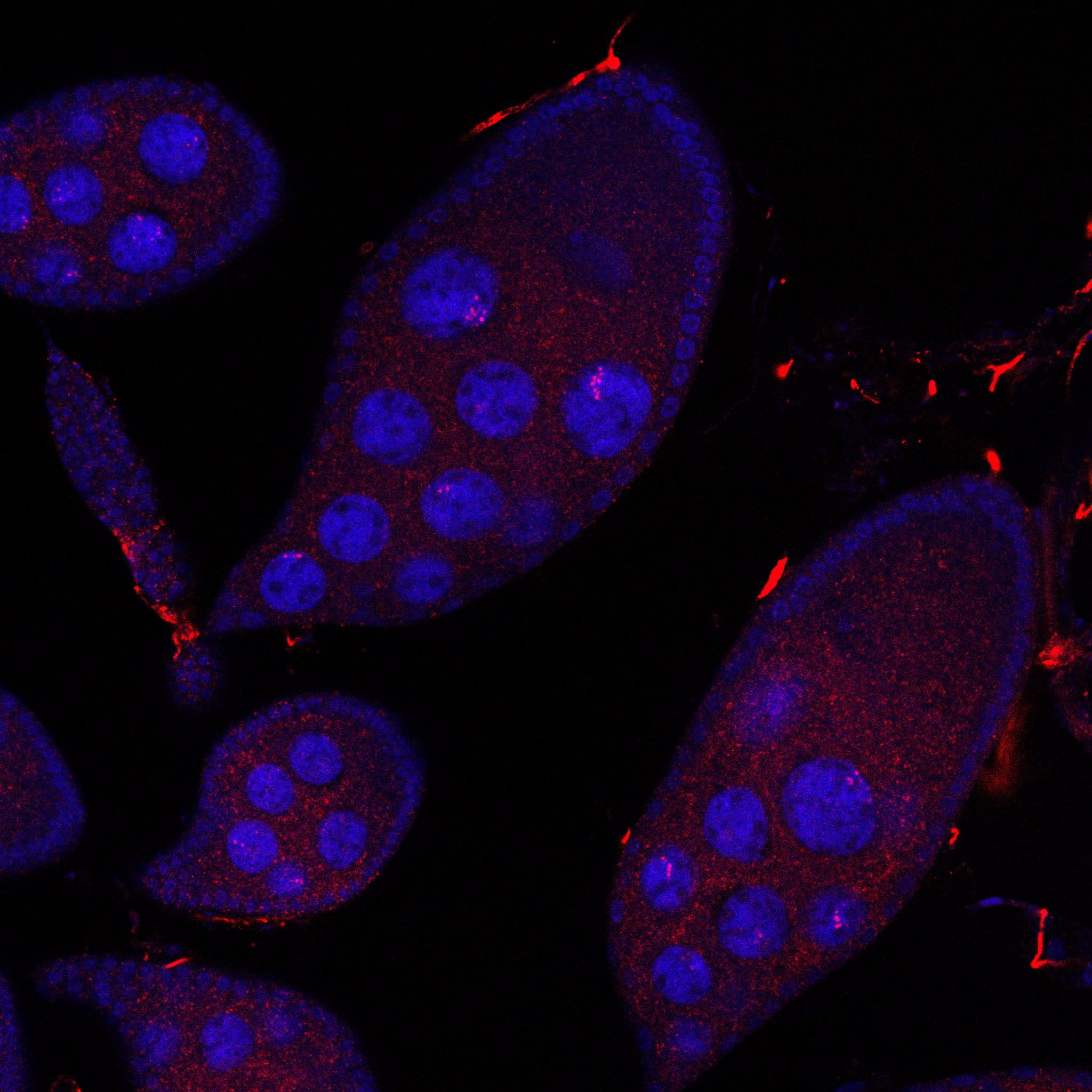

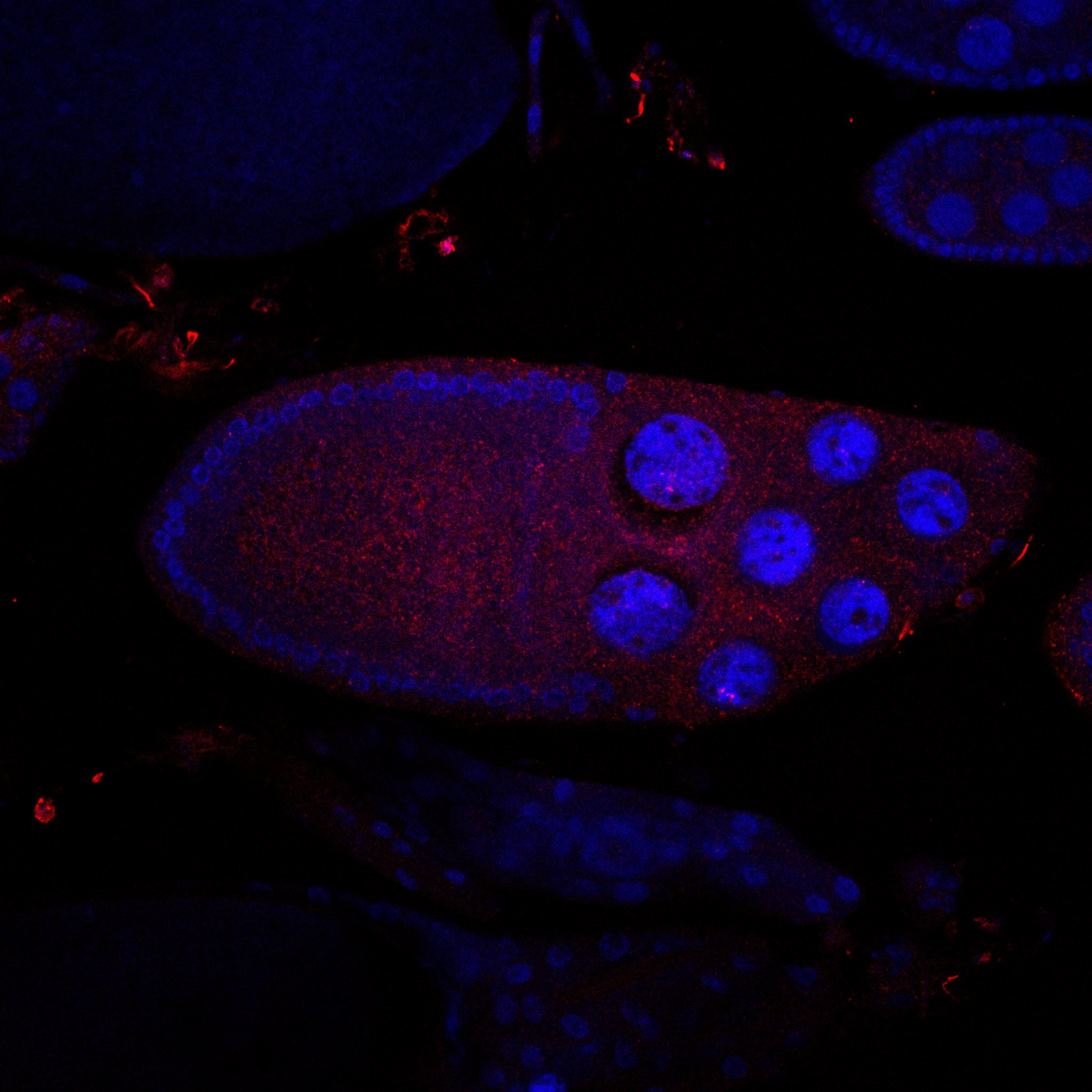
***


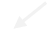

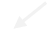


**Hybrids*
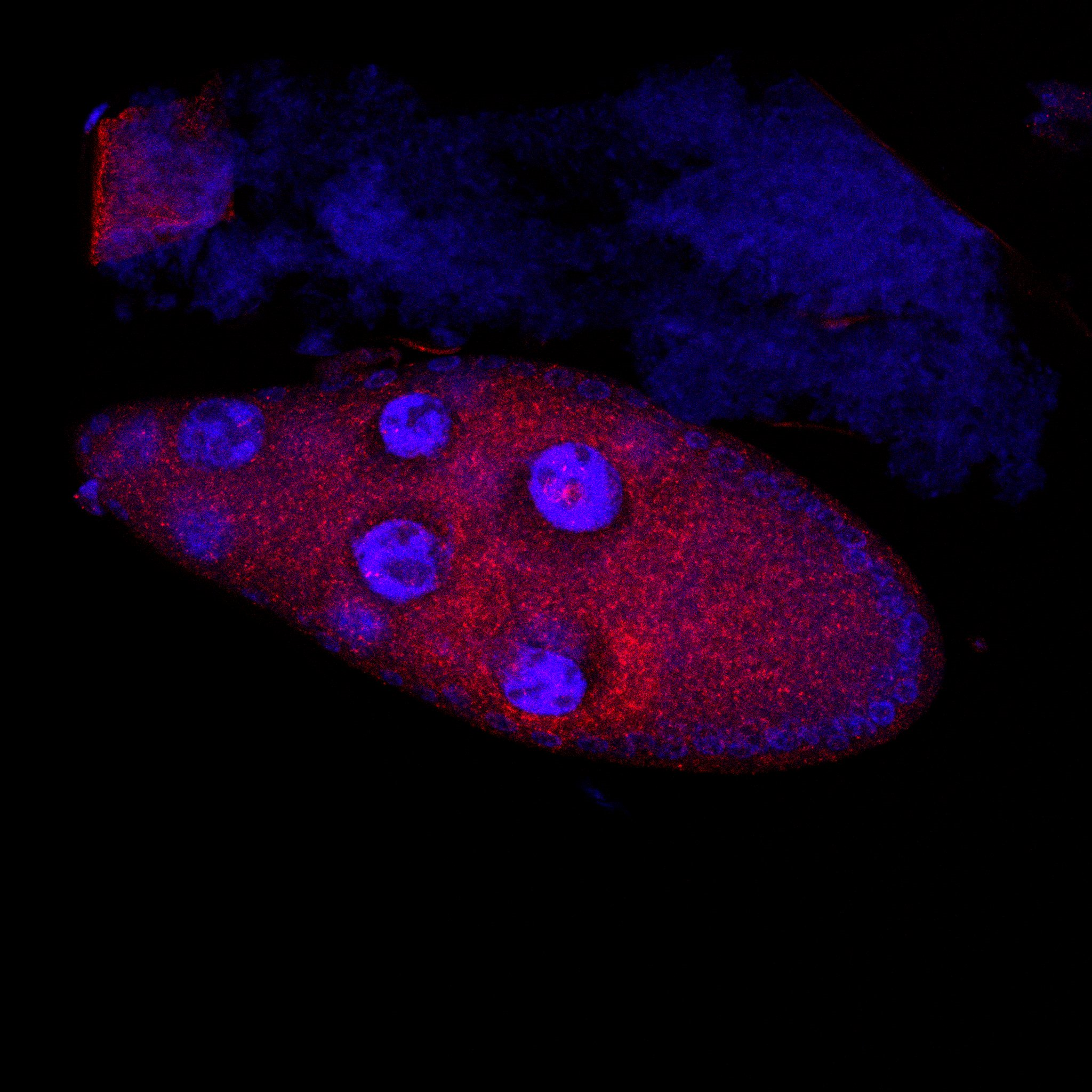
***


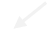


***piwi***

***D. buzzatii D. koepfeare***

***
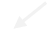

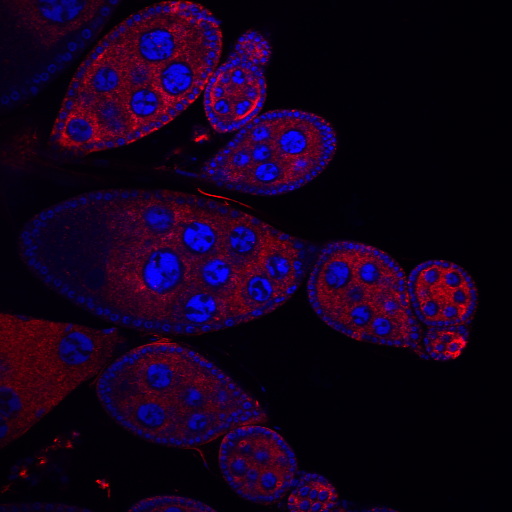

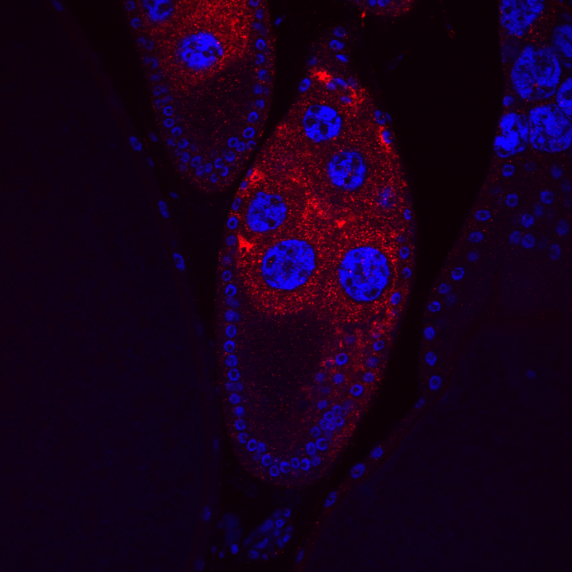
***


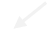


***
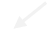
***
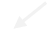


**Hybrids**


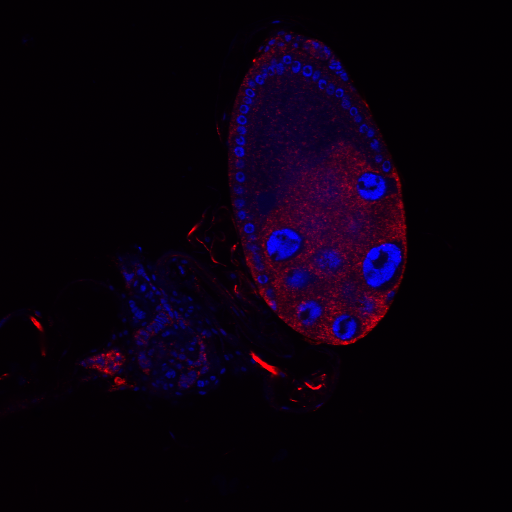


***
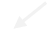
***
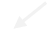


***rhino***

***
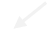
D. buzzatii D. koepfeare***


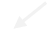

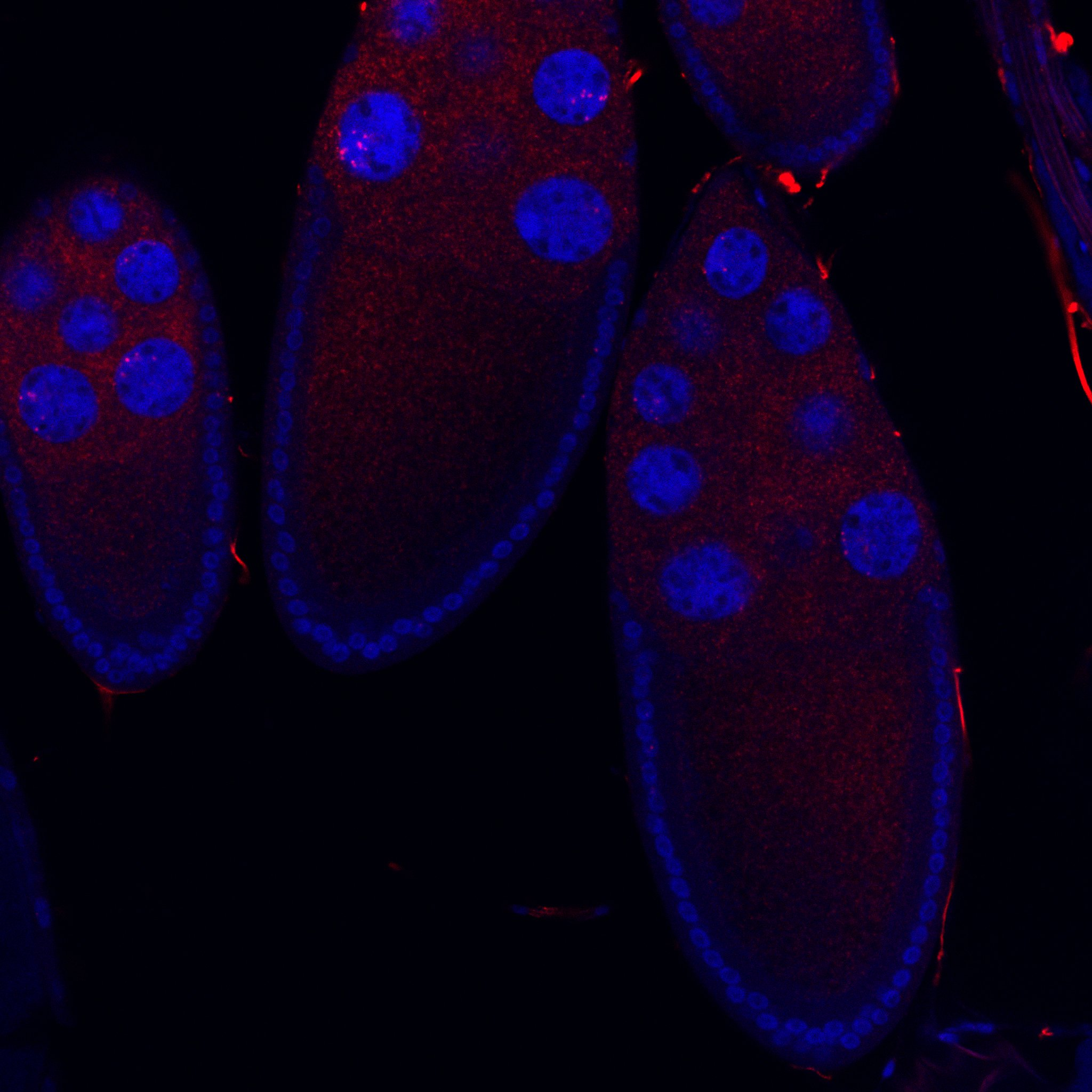

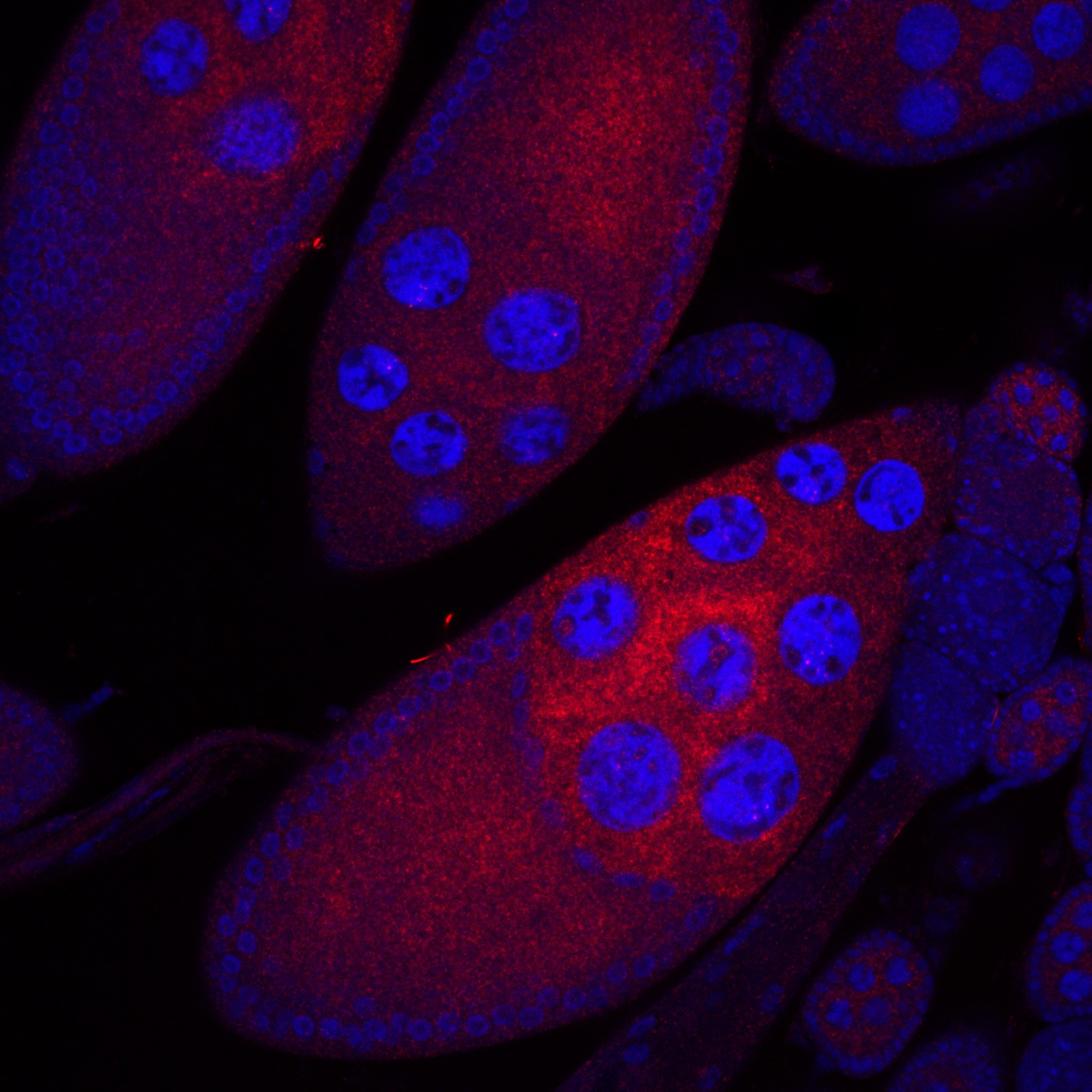


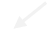


**Hybrids**
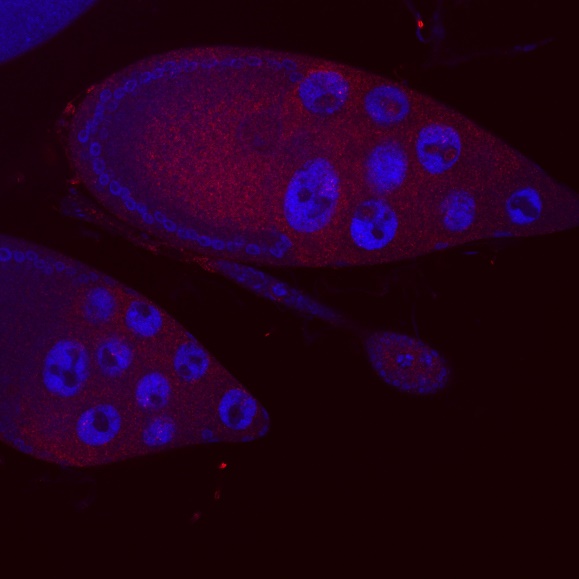


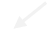


***spindle E***

***
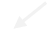
D. buzzatii D. koepfeare***


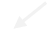

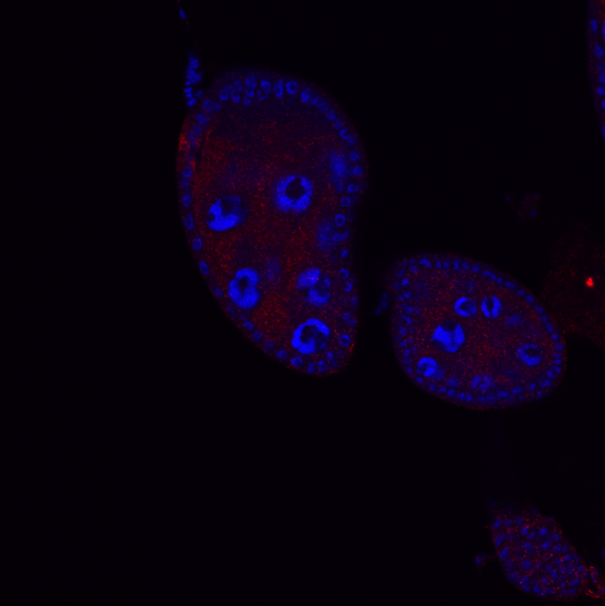

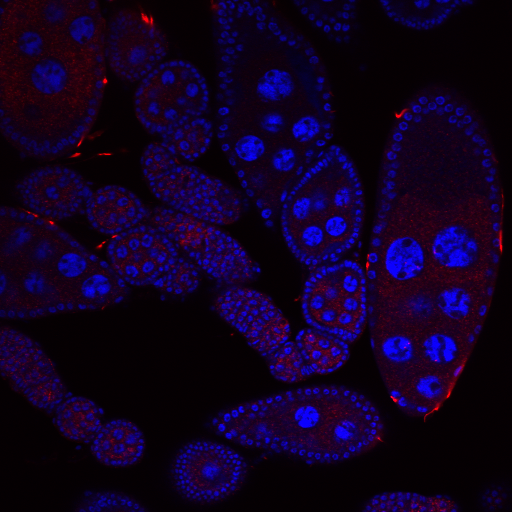

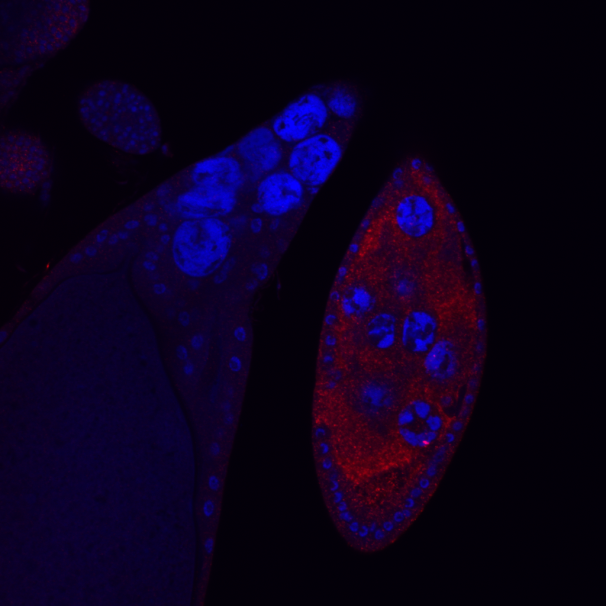


**Hybrids**

***
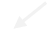
***
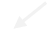


***zucchini***

***
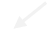
D. buzzatii D. koepfeare***


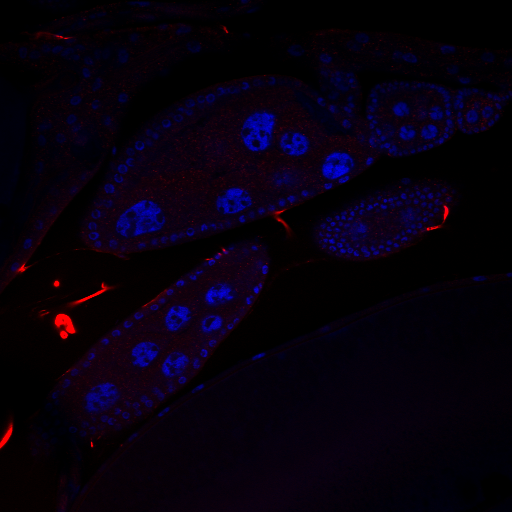

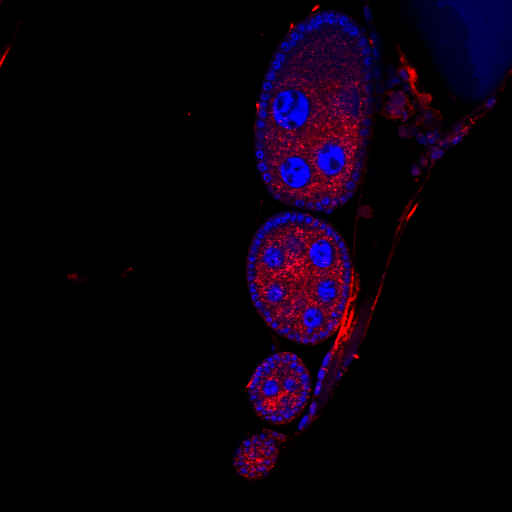


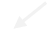

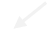

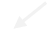


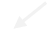

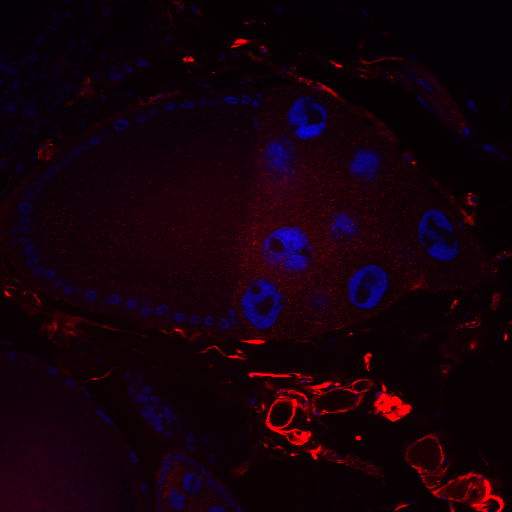


**Hybrids**
