# Supplementary material for: Drosophila Interspecific Hybridization Causes a Deregulation of the piRNA Pathway Genes
Source: Genes (Basel). 2020 Feb 19;11(2):215. doi: 10.3390/genes11020215 (PMC7073935; doi:10.3390/genes11020215)
Supplement: Supplementary file 1 [file genes-11-00215-s001.zip › supplementary/Supplementary_file_3.docx]

|  | **Species** | transcript (bp) | aminoacids | exons |
| --- | --- | --- | --- | --- |
| **ago3** | *D. mojavensis* | 2734 | 907 | 2 |
| **armi** | *D. mojavensis* | 3708 | 1170 | 5 |
| **aub** | *D. mojavensis* | 3075 | 850 | 9 |
| **krimp** | *D. mojavensis* | 2830 | 871 | 2 |
| **mt2** | *D. mojavensis* | 1005 | 334 | 2 |
| **piwi** | *D. mojavensis* | 2809 | 881 | 8 |
| **rhino** | *D. mojavensis* | 1593 | 530 | 2 |
| **spnE** | *D. mojavensis* | 4487 | 1431 | 11 |
| **zuc** | *D. mojavensis* | 894 | 237 | 1 |

**Supplementary file 3A**: piRNA pathway genes features in *D. mojavensis*, the closest relative to *D. buzzatii* and *D. koepferae* with a sequenced genome.

| ***D. melanogaster*** |  |  |  |
| --- | --- | --- | --- |
|  | transcript (bp) | aminoacids | exons |
| **ago3** | 2800 | 867 | 6 |
| **armi** | 4134 | 1188 | 6 |
| **aub** | 2825 | 866 | 9 |
| **krimp** | 2549 | 746 | 2 |
| **mt2** | 1038 | 345 | 2 |
| **piwi** | 3073 | 843 | 8 |
| **rhino** | 1257 | 418 | 2 |
| **spnE** | 4678 | 1434 | 11 |
| **zuc** | 1148 | 253 | 1 |

**Supplementary file 3B:** piRNA pathway genes main feature in the genetic model *D. melanogaster*.

| ***D. koepferae*** |  |  |  |  |  |  |
| --- | --- | --- | --- | --- | --- | --- |
| Name | [From](../../../../C:/censor/help.html#MAP-FROMTO) | [To](../../../../C:/censor/help.html#MAP-FROMTO) | [Class](../../../../C:/censor/help.html#MAP-CLASS) | [Dir](../../../../C:/censor/help.html#MAP-DIR) | [Sim](../../../../C:/censor/help.html#MAP-SIM) | [Score](../../../../C:/censor/help.html#MAP-SCORE) |
| [Helitron-2_EPa](../../../../C:/protected/repbase_extract.php%3Faccess=Helitron-2_EPa&format=EMBL) | 3069 | 3223 | DNA/Helitron | d | 0.7431 | 203 |
| [Helitron-1N1_DVir](../../../../C:/protected/repbase_extract.php%3Faccess=Helitron-1N1_DVir&format=EMBL) | 1 | 168 | DNA/Helitron | d | 0.8012 | 788 |
| [Homo6](../../../../C:/protected/repbase_extract.php%3Faccess=Homo6&format=EMBL) | 784 | 891 | DNA/hAT | d | 0.7664 | 354 |
| [Homo6](../../../../C:/protected/repbase_extract.php%3Faccess=Homo6&format=EMBL) | 619 | 671 | DNA/hAT | d | 0.8302 | 320 |
| [Homo6](../../../../C:/protected/repbase_extract.php%3Faccess=Homo6&format=EMBL) | 1112 | 1206 | DNA/hAT | d | 0.7895 | 436 |
| [Gypsy-14_LH-I](../../../../C:/protected/repbase_extract.php%3Faccess=Gypsy-14_LH-I&format=EMBL) | 1739 | 1797 | LTR/Gypsy | d | 0.8182 | 234 |
| [Mariner-2_DAn](../../../../C:/protected/repbase_extract.php%3Faccess=Mariner-2_DAn&format=EMBL) | 1730 | 2142 | DNA/Mariner | d | 0.7210 | 1396 |
| [KolobokH-1A_RIr](../../../../C:/protected/repbase_extract.php%3Faccess=KolobokH-1A_RIr&format=EMBL) | 5359 | 5412 | DNA/Kolobok | c | 0.7963 | 206 |
|  |  |  |  |  |  |  |
|  |  |  |  |  |  |  |
| ***D. mojavensis*** |  |  |  |  |  |  |
| [Name](https://www.girinst.org/censor/help.html#MAP-NAME) | [From](https://www.girinst.org/censor/help.html#MAP-FROMTO) | [To](https://www.girinst.org/censor/help.html#MAP-FROMTO) | [Class](https://www.girinst.org/censor/help.html#MAP-CLASS) | [Dir](https://www.girinst.org/censor/help.html#MAP-DIR) | [Sim](https://www.girinst.org/censor/help.html#MAP-SIM) | [Score](https://www.girinst.org/censor/help.html#MAP-SCORE) |
| [Helitron1_Dmoj](https://www.girinst.org/protected/repbase_extract.php?access=Helitron1_Dmoj&format=EMBL) | 294 | 328 | DNA/Helitron | d | 0.8571 | 233 |
| [Helitron1_Dmoj](https://www.girinst.org/protected/repbase_extract.php?access=Helitron1_Dmoj&format=EMBL) | 458 | 579 | DNA/Helitron | d | 0.8443 | 695 |
| [Homo11](https://www.girinst.org/protected/repbase_extract.php?access=Homo11&format=EMBL) | 43 | 137 | DNA/hAT | d | 0.8372 | 473 |
| [Homo11](https://www.girinst.org/protected/repbase_extract.php?access=Homo11&format=EMBL) | 2022 | 2291 | DNA/hAT | d | 0.9387 | 1981 |
| [DNAREP1_DSim](https://www.girinst.org/protected/repbase_extract.php?access=DNAREP1_DSim&format=EMBL) | 56 | 212 | DNA/Helitron | d | 0.7838 | 634 |
| [BEL-8_DMo-LTR](https://www.girinst.org/protected/repbase_extract.php?access=BEL-8_DMo-LTR&format=EMBL) | 207 | 461 | LTR/BEL | c | 0.8902 | 1649 |
| [Galileo_DB](https://www.girinst.org/protected/repbase_extract.php?access=Galileo_DB&format=EMBL) | 403 | 446 | DNA/P | c | 0.8182 | 255 |
| [Homo4](https://www.girinst.org/protected/repbase_extract.php?access=Homo4&format=EMBL) | 251 | 337 | DNA/hAT | c | 0.8046 | 477 |
| [Helitron-N1_DVir](https://www.girinst.org/protected/repbase_extract.php?access=Helitron-1N1_DVir&format=EMBL) | 1 | 154 | DNA/Helitron | d | 0.8400 | 813 |
| [Helitron-1_DVir](https://www.girinst.org/protected/repbase_extract.php?access=Helitron-1_DVir&format=EMBL) | 1 | 26 | DNA/Helitron | d | 0.9231 | 204 |
| [Helitron-N1_DBi](https://www.girinst.org/protected/repbase_extract.php?access=Helitron-N1_DBi&format=EMBL) | 26 | 246 | DNA/Helitron | d | 0.7534 | 943 |
| [Homo6](https://www.girinst.org/protected/repbase_extract.php?access=Homo6&format=EMBL) | 1144 | 1217 | DNA/hAT | d | 0.7600 | 309 |

**Supplementary file 3C:** TE sequences in the first intron of the *ago3* gene in *D. koepferae* and *D. mojavensis.* Dir: direct (d) or complementary (c) sequence. Sim: sequence similarity. Score: score of the alignment. No TE sequences were found in the corresponding *D. buzzatii* intron.
